# Rosmarinic Acid, a Rosemary Extract Polyphenol, Increases Skeletal Muscle Cell Glucose Uptake and Activates AMPK

**DOI:** 10.3390/molecules22101669

**Published:** 2017-10-07

**Authors:** Filip Vlavcheski, Madina Naimi, Brennan Murphy, Tomas Hudlicky, Evangelia Tsiani

**Affiliations:** 1 Department of Health Sciences, Brock University, St. Catharines, ON L2S 3A1, Canada; fv11vi@brocku.ca (F.V.); madinanaimi@gmail.com (M.N.); 2 Department of Chemistry, Brock University, St. Catharines, ON L2S 3A1, Canada; murphyb2013@gmail.com (B.M.); thudlicky@brocku.ca (T.H.); 3 Centre for Bone and Muscle Health, Brock University, St. Catharines, ON L2S 3A1, Canada

**Keywords:** muscle, rosmarinic acid, AMPK, glucose uptake

## Abstract

Skeletal muscle is a major insulin-target tissue and plays an important role in glucose homeostasis. Impaired insulin action in muscles leads to insulin resistance and type 2 diabetes mellitus. 5′ AMP-activated kinase (AMPK) is an energy sensor, its activation increases glucose uptake in skeletal muscle and AMPK activators have been viewed as a targeted approach in combating insulin resistance. We previously reported AMPK activation and increased muscle glucose uptake by rosemary extract (RE). In the present study, we examined the effects and the mechanism of action of rosmarinic acid (RA), a major RE constituent, in L6 rat muscle cells. RA (5.0 µM) increased glucose uptake (186 ± 4.17% of control, *p* < 0.001) to levels comparable to maximum insulin (204 ± 10.73% of control, *p* < 0.001) and metformin (202 ± 14.37% of control, *p* < 0.001). Akt phosphorylation was not affected by RA, while AMPK phosphorylation was increased. The RA-stimulated glucose uptake was inhibited by the AMPK inhibitor compound C and was not affected by wortmannin, an inhibitor of phosphoinositide 3-kinase (PI3K). The current study shows an effect of RA to increase muscle glucose uptake and AMPK phosphorylation. RA deserves further study as it shows potential to be used as an agent to regulate glucose homeostasis.

## 1. Introduction

Skeletal muscle is a primary target tissue of insulin and plays a critical role in the maintenance of glucose homeostasis [1]. After binding to its receptor, insulin increases the receptor tyrosine kinase activity which leads to GLUT4 glucose transporter translocation to the plasma membrane via activation of the lipid kinase phosphoinositide 3-kinase (PI3K) and the serine/threonine kinase Akt/PKB [2,3]. Impairment of the PI3K–Akt cascade leads to insulin resistance and type 2 diabetes mellitus (T2DM) [4,5,6], a disease expected to affect 412 million people globally by 2040 [7].

AMP-activated protein kinase (AMPK) is a serine/threonine kinase that has a potential to regulate blood glucose levels. As an energy sensor, AMPK is activated by increased AMP/ATP ratio and/or via activation of its upstream kinases, liver kinase B1 (LKB1) and calmodulin dependent protein kinases (CaMKKs) [8,9]. Muscle AMPK is activated through muscle contraction/exercise [8]. Several compounds including metformin [10], thiazolidineones [11] and polyphenols such as resveratrol [12] and naringenin [13] are also known to activate AMPK and increase muscle glucose uptake. In recent years, AMPK activators have been recognized as a promising pharmacological intervention for the prevention and treatment of T2DM [8,9,14,15,16,17,18].

Rosemary (*Rosmarinus officinalis* L.) is an aromatic evergreen plant endemic to the Mediterranean region and South America that is reported to exhibit antioxidant, anticancer and antimicrobial effects [19,20]. In addition, beneficial effects have been reported in regards to lipid metabolism and plasma glucose levels [21,22,23,24,25,26]. Previous studies by our group examined the effects of rosemary extract (RE) [27] on skeletal muscle cells and found a significant increase in glucose uptake and AMPK activation. In vivo studies demonstrated that administration of RE decreased plasma glucose levels in streptozotocin-induced diabetic mice [21], rats [23,25,26], alloxan-induced diabetic rabbits [22], and in genetic [24] and dietary [26,28,29] animal models of obesity and insulin resistance. RE is composed of various polyphenols with carnosic acid (CA) and rosmarinic acid (RA) being the most abundant in regards to concentration [30]. It is therefore possible that the beneficial effects observed with RE administration may be due to the action of a specific polyphenol. We recently found a significant increase in muscle cell glucose uptake and activation of AMPK by CA [31].

In the present study, we focused on RA and examined its direct effect on muscle cell glucose uptake, and investigated the signaling molecules that may be involved.

## 2. Results

### 2.1. Rosmarinic Acid (RA) Stimulates Muscle Cell Glucose Uptake

**Figure 1:**
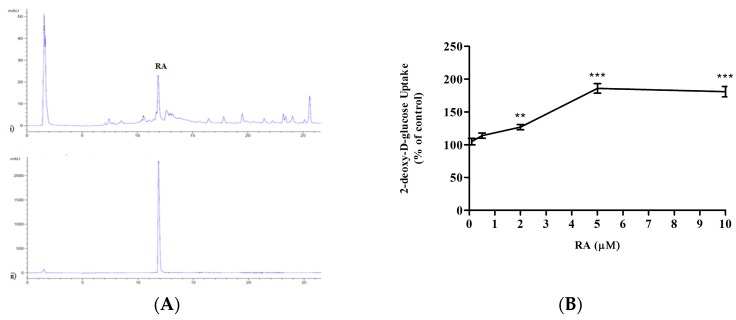
(**A**) Levels of rosmarinic acid (RA) present in rosemary extract (RE). High-performance liquid chromatography (HPLC) chromatograph representing RE (upper panel) and RA standard (lower panel). (**B**) Effects of rosmarinic acid (RA) on glucose uptake (dose response). Serum-deprived L6 myotubes were incubated without or with 0.1, 0.5, 2, 5 or 10 μM RA for 4 h. 2-deoxy-d-glucose uptake was measured according to the methods. Data are expressed as percentage of control. Results are the mean ± SE of 3–4 independent experiments. ** *p* < 0.01, *** *p* < 0.001, vs. control.

We reported previously that glucose uptake was significantly increased in L6 muscle cells by 5 μg/mL of RE [27]. Additionally, previous studies have indicated that RA is one of the major constituents found in RE [30], and therefore we examined the levels of RA present in the RE that was extracted in our lab and utilized previously [27]. To this end, we performed high-performance liquid chromatography (HPLC) and a representative chromatograph is shown in Figure 1A. The retention time of the peak which corresponds to RA from the standard was utilized to determine the presence of RA in the extract. The area under the peak corresponding to the RA present in the extract was used to quantify the relative amount of RA. Our data demonstrate that RE contained 13.39 ± 0.23% RA. Based on these values and the molecular weight of RA (MW: 360.13 g/mol), we calculated the concentration of RA in media containing 5 μg/mL of RE, a concentration that elicited maximal stimulation of glucose uptake in our previous study [27], and found that the corresponding concentration of RA is 2.0 μM. We then went on to investigate whether RA at a concentration of 2.0 μM would have any effect on the glucose uptake. However, we wished to obtain a dose-response curve and for this reason we used additional concentrations.

L6 muscle cells were differentiated in α-Minimal Essential Medium (α-MEM) containing 2% (*v*/*v*) Fetal bovine serum (FBS), as previously described [12,13,27]. Myotubes were incubated with 0.1, 0.5, 2, 5 or 10 μM RA for 4 h (Figure 1B). RA at 0.1 and 0.5 μM did not increase glucose uptake (105 ± 4.80% of control and 114 ± 3.96% of control respectively, both *p* > 0.05). However, higher concentration of RA resulted in a dose-dependent increase in glucose uptake. Significant stimulation of glucose uptake was seen at 2 μM RA (127 ± 4.04% of control, *p* < 0.01), and maximum stimulation was seen at 5 μM RA (186 ± 7.31% of control, *p* < 0.001) (Figure 1). It should be noted that higher concentration of RA (10 μM) also stimulated glucose uptake (181 ± 7.88% of control, *p* < 0.001) without any changes in cell morphology or cell toxicity assessed by microscopic examination.

**Figure 2:**
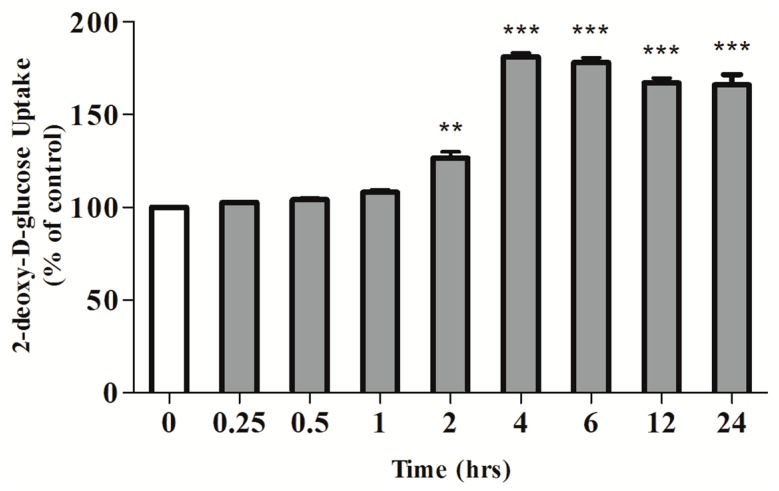
Effects of rosmarinic acid (RA) on glucose uptake (Time-course). Serum-deprived L6 myotubes were incubated without or with 5 μM RA for the indicated time. Data are expressed as percentage of control. Results are the mean ± SE of 3–5 independent experiments. ** *p* < 0.01, *** *p* < 0.001, vs control.

We further investigated if the effect of RA on glucose uptake is time-dependent. Fully differentiated myotubes were incubated with 5 μM RA for 0.25, 0.5, 1, 2, 4, 6, 12 or 24 h (Figure 2). Significant stimulation was seen after 2 h of RA exposure (126.6 ± 3.32% of control, *p* < 0.01) while maximum stimulation was observed after 4 h of exposure (186 ± 7.38% of control, *p* < 0.001) (Figure 2). Longer exposure time of 12 and 24 h to RA also significantly stimulated glucose uptake (166 ± 4.00% of control and 167 ± 2.00% of control, respectively, both *p* < 0.001) (Figure 2).

**Figure 3:**
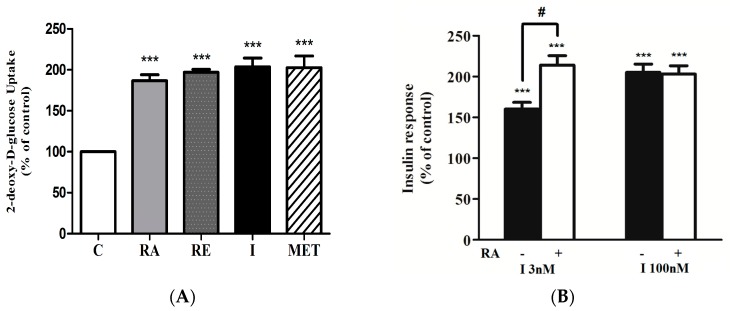
(**A**) Effects of rosmarinic acid (RA), rosemary extract (RE), insulin (I) and metformin (MET) on glucose uptake. Serum-deprived L6 myotubes were incubated without (control, C) or with 5 µM RA (4 h), 5 µg/mL RE (4 h), 100 nM I (0.5 h) or 2 mM MET (2 h) followed by 2-deoxy-d-glucose uptake measurements. Results are the mean ± SE of 5 independent experiments. *** *p* < 0.001, vs control. (**B**) Effect of rosmarinic acid (RA) on insulin-stimulated glucose uptake. Serum-deprived L6 myotubes were incubated without or with 5 μM RA (4 h) followed by stimulation with 3 nM or 100 nM insulin for 0.5 h and glucose uptake measurements. Results are the mean ± SE of 3–4 independent experiments. *** *p* < 0.001, vs. control, ^#^ *p* < 0.05 vs. insulin alone.

We compared the effect of RA with that of insulin and metformin, the most widely used/prescribed medication for T2DM. It is important to note that the maximum stimulation of glucose uptake seen with RA treatment (186 ± 7.31% of control, *p* < 0.001) was comparable to the response seen with maximum insulin (100 nM, 0.5 h, 204 ± 10.73% of control, *p* < 0.001) and metformin (2 mM, 2 h, 202 ± 14.37% of control, *p* < 0.001) stimulation (Figure 3A). Exposure of myotubes to 5 μg/mL of rosemary extract (RE) also significantly increased glucose uptake (197 ± 3.60% of control, *p* < 0.001) in agreement with our previous studies [27,31].

### 2.2. Effect of Rosmarinic Acid (RA) on Insulin-Stimulated Glucose Uptake

We further investigated the effects of RA on insulin-stimulated glucose uptake. Myotubes were exposed to 5.0 μM RA (4 h) followed by treatment with 3 nM (sub-maximal) or 100 nM (maximal) insulin. Insulin at submaximal 3 nM concentration increased glucose uptake (I 3 nM: 160 ± 8.34% of control); and this response was significantly enhanced by RA treatment (RA + I 3 nM: 214 ± 11.49% of control) (Figure 3B). However, RA did not affect the maximum insulin-stimulated glucose uptake (I 100 nM: 205 ± 10.34% of control, RA + I 100 nM: 203 ± 10.17% of control) (Figure 3B).

### 2.3. Effects of Rosmarinic Acid (RA) on AMPK Signaling

**Figure 4:**
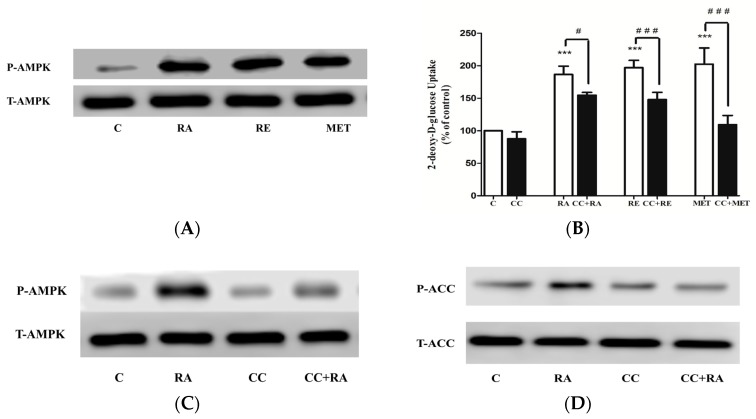
(**A**) Effect of RA on AMPK. L6 myotubes were treated without (control, C) or with 5.0 μM RA (2 h), 5 μg/mL of RE (2 h), 2 mM metformin (MET) (2 h). Whole-cell lysates were prepared, resolved by SDS-PAGE and immunoblotted with specific antibodies that recognize phospho-AMPK (Thr 172) (P-AMPK) or total AMPK (T-AMPK). (**B**) Effect of compound C (CC) on rosmarinic acid (RA)-induced glucose uptake. Cells were incubated in the absence or presence of 25 μM compound C (CC) for 30 min followed by the addition of 5.0 μM RA, 5 μg/mL of RE or 2 mM MET and glucose uptake measurements. Results are the mean ± SE of 6–7 independent experiments. *** *p* < 0.001 vs control. ^###^ *p* < 0.001 vs treatment in the absence of CC. (**C**) Effect of compound C (CC) on RA-induced AMPK and (**D**) ACC phosphorylation. Whole-cell lysates were immunoblotted for phospho-AMPK (Thr 172) (P-AMPK), total AMPK (T-AMPK) (**C**) or for phospho-ACC (Ser 79) (P-ACC) or total ACC (T-ACC) (**D**).

In our previous studies we found significant increase in AMPK phosphorylation by RE [27] and carnosic acid [31], and therefore we hypothesize that RA may also increase AMPK phosphorylation. Treatment of L6 myotubes with RA resulted in a robust increase in AMPK phosphorylation on threonine 172 [32], an indicator of activation (Figure 4A). Importantly, the activation of AMPK seen with RA treatment was at the same level as with 2mM metformin treatment (Figure 4A). Treatment with RE also increased AMPK phosphorylation (Figure 4A) in agreement with our previous studies [27,31]. To examine the involvement of AMPK in glucose uptake, we used the AMPK inhibitor, compound C (CC). RA-stimulated glucose uptake was significantly inhibited by CC (RA: 186 ± 4.17% of control, CC + RA: 163 ± 8.23% of control) (Figure 4B). RE-stimulated glucose uptake was also inhibited by CC (RE: 197 ± 3.60% of control, CC + RE: 135 ± 4.01% of control) (Figure 4B), while the metformin-stimulated glucose uptake was abolished in the presence of CC (MET: 202 ± 14.37% of control, CC + MET: 103 ± 7.23% of control) (Figure 4B). We also investigated the effect of CC on RA-stimulated phosphorylation of AMPK (Figure 4C). CC blocked the RA-induced AMPK phosphorylation, indicating that the use of a CC inhibitor in our study was effective in blocking AMPK (Figure 4C). Additionally, we examined the phosphorylation of the downstream physiological target of AMPK, acetyl-CoA carboxylase (ACC). Treatment with RA resulted in significant increase in ACC phosphorylation that was completely blocked in the presence of CC (Figure 4D), further indicating AMPK activation by RA treatment and effective blocking by CC.

**Figure 5:**
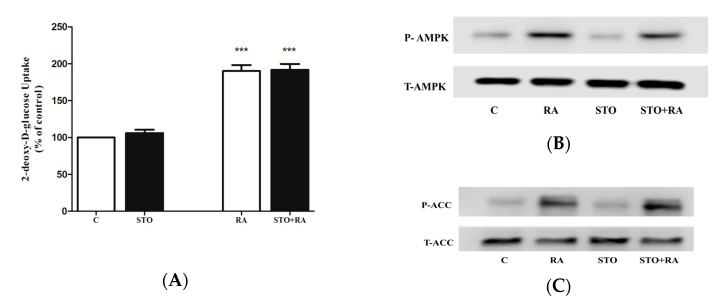
(**A**) Effect of STO-609 on rosmarinic acid (RA)-induced glucose transport. L6 myotubes were incubated in the absence (control, C) or presence of 27 μM STO-609 (STO) for 1 hour followed by the addition of 5.0 μM RA (4 h) and glucose uptake measurements. Results are the mean ± SE of 3 independent experiments. *** *p* < 0.001 vs. control. (**B**) Effect of STO-609 (STO) on AMPK and (**C**) ACC phosphorylation. Whole-cell lysates were immunoblotted for phospho (Thr 172) (P-AMPK) or total AMPK (T-AMPK) (**B**) or for phospho (Ser 79) (P-ACC) or total ACC (T-ACC) (**C**).

To examine the involvement of Ca^2+^/calmodulin-dependent kinase kinase (CaMKK)—an upstream regulator of AMPK—in RA-stimulated glucose uptake, we used the CaMKK-selective inhibitor STO-609 [33]. Exposure of L6 myotubes to STO-609 (27 μM) did not affect the basal glucose uptake (106 ± 2.6% of control) (Figure 5A). Furthermore, the RA-stimulated glucose uptake was not affected by STO-609 (RA: 190 ± 4.6% of control, STO + RA: 191 ± 4.7% of control) (Figure 5A). Additionally, we investigated the effect of STO-609 on RA-induced phosphorylation of AMPK and its downstream effector ACC (Figure 5B,C). Treatment with STO-609 alone did not affect AMPK or ACC phosphorylation. Furthermore, the RA-induced phosphorylation of AMPK and ACC was not affected by STO-609 treatment, indicating that CaMKK is not involved in the RA-induced phosphorylation of AMPK and ACC (Figure 5B,C).

### 2.4. Effect of Rosmarinic Acid (RA) on PI3K–Akt Signaling Cascade

**Figure 6:**
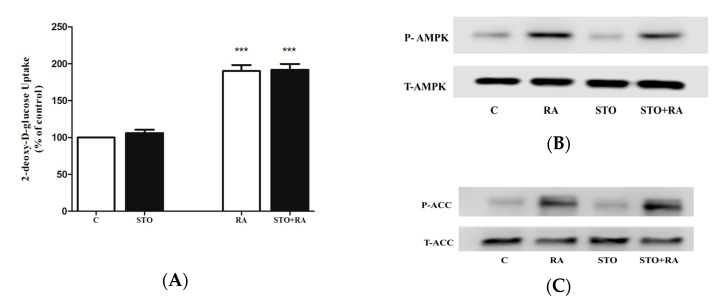
(**A**) Effect of wortmannin (W) on rosmarinic acid (RA)-induced glucose uptake. L6 myotubes were incubated in the absence (control, C) or presence of 100 nM wortmannin (W) for 15 min followed by the addition of 5.0 μM RA (4 h), 5 μg/mL of RE (4 h) or 100 nM insulin (30 min) and glucose uptake measurements. Results are the mean SE of 6–7 independent experiments. *** *p* < 0.001 vs. control. ^###^ *p* < 0.001 vs. treatment in the absence of wortmannin. (**B**) and (**C**) Effect of rosmarinic acid (RA) on Akt and P70 S6K phosphorylation. L6 myotubes were treated without (control, C) or with 5.0 μM RA (2 h), I 100 nM (30 min) or 2 mM metformin (MET) (2 h). Whole-cell lysates were prepared, resolved by SDS-PAGE and immunoblotted with specific antibodies that recognize phosphorylated Akt (P-Akt) or total Akt (T-Akt) (**B**) or phosphorylated p70 S6K (P-p70S6K) or total p70 S6K (T-p70S6K) (**C**).

The effects of RA on the PI3K-Akt signaling cascade, the key players involved in insulin-stimulated glucose uptake, were also examined. For that purpose, we used the PI3K inhibitor wortmannin. Wortmannin did not have an effect on RA-stimulated glucose uptake (RA: 186 ± 4.17% of control, W + RA: 182 ± 6.32% of control) or RE-stimulated glucose uptake (RE: 202 ± 10.60% of control, W + RE: 192 ± 10.42% of control), indicating that the PI3K signaling is not involved (Figure 6A). On the other hand, wortmannin completely blocked insulin-stimulated glucose uptake (I: 204 ± 9.10% of control, W + I: 123 ± 8.91% of control), indicating effective blocking of PI3K by wortmannin. We further examined the effect of RA on Akt. Our data indicated that RA does not have an effect on Akt phosphorylation or expression, contrary to the significant phosphorylation of Akt observed with insulin stimulation (Figure 6B). Metformin also did not affect the levels of Akt phosphorylation/expression (Figure 6B). In addition, the phosphorylation of the downstream physiological target of Akt, p70 S6K, was investigated. Treatment with RA and metformin did not affect the phosphorylation/expression of p70 S6K. In contrast, treatment with insulin lead to a robust increase in p70 S6K phosphorylation (Figure 6C). Altogether, these data show no effect of RA on the PI3K-Akt signaling cascade.

### 2.5. Effect of PKC Inhibition on RA-Stimulated Glucose Uptake

**Figure 7:**
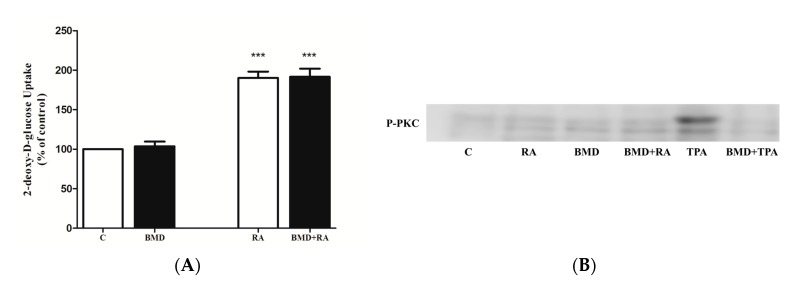
(**A**) Effect of bisindolylmaleimide I (BMD) on rosmarinic acid (RA)-induced glucose transport. L6 myotubes were incubated in the absence (control, C) or presence of 1 μM bisindolylmaleimide I (BMD) for 1 h followed by the addition of 5.0 μM RA (4 h) and glucose uptake measurements. Results are the mean ± SE of 3 independent experiments. *** *p* < 0.001 vs. control. (**B**) Effect of RA on PKC phosphorylation. Cells were incubated in the absence or presence of 1 μM BMD for 1 h followed by addition of 5.0 μM RA (2 h) or 200 nM 12-*O*-tetradecanoylphorbol-13-acetate (TPA) (20 min). Whole-cell lysates were prepared, resolved by SDS-PAGE and immunoblotted with specific antibodies that recognize phosphorylated/activated PKC.

Previous evidence indicates that activation of protein kinase C (PKC) leads to an increase in glucose uptake in muscle cells in response to different stimuli [34,35,36,37]. Based on this evidence and our data showing partial and not complete inhibition of RA-stimulated glucose uptake with AMPK inhibition (Figure 4B), we hypothesized that PKCs may play a role in RA-stimulated glucose uptake. To address this hypothesis, we used bisindolylmaleimide I (BMD), a selective inhibitor of PKCα-, β_I_-, β_II_-, γ-, δ- and ε- isozymes, used extensively in other studies [38,39,40]. BMD did not have any effect on the basal glucose uptake (103 ± 3.4% of control) (Figure 7A). Furthermore, RA-stimulated glucose uptake was not affected by BMD (RA: 190 ± 4.6% of control, BMD + RA: 188 ± 7.3% of control) (Figure 7A). Additionally, we investigated the effect of RA on PKC phosphorylation, which is correlated with an increase in PKC activity [41], using a specific antibody phospho-PKC (pan) that recognizes conventional (α, β_I_, β_II_) and novel (δ, ε, η and θ) PKCs (Figure 7B). Treatment of the cells with RA did not have any effect on PKC phosphorylation/activation (Figure 7B). On the other hand, treatment with 12-*O*-tetradecanoylphorbol-13-acetate (TPA) significantly increased PKC phosphorylation. Furthermore, in the presence of BMD, the TPA-induced PKC phosphorylation/activation was completely abolished, indicating that in our experiments, BMD acts as an effective inhibitor of PKCs.

### 2.6. Effect of RA on Glucose Transporters

**Figure 8:**
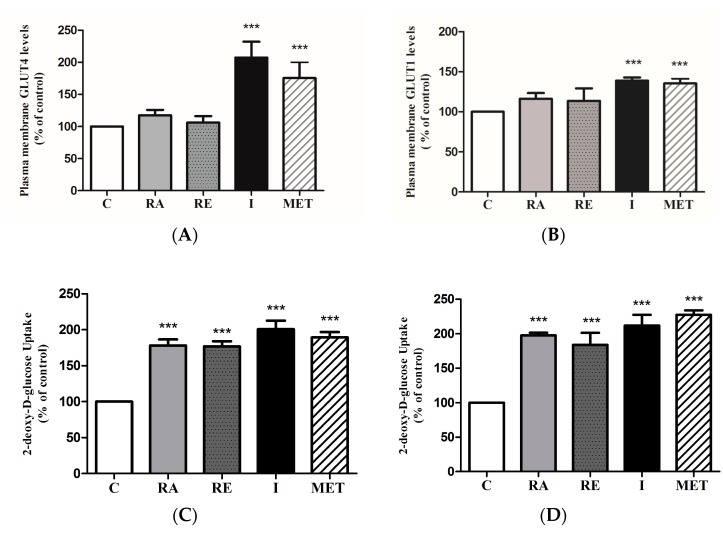
Effect of RA on plasma membrane GLUT levels and glucose uptake in GLUT-overexpressing cells. L6 GLUT4myc (**A**,**C**) or GLUT1myc (**B**,**D**) overexpressing cells were treated with 5 µM rosmarinic acid (RA) (4 h), 5 μg/mL rosemary extract (RE) (4 h), insulin (I) (100 nM, 0.5 h), or 2 mM metformin (MET) (4 h), followed by GLUT4 or GLUT1 plasma membrane transporter level measurements (**A**,**B**) or glucose uptake measurements (**C**,**D**). Results are mean ± SE of 5–7 independent experiments. *** *p* < 0.001 vs. control.

To elucidate the mechanism by which RA increased glucose uptake, we measured plasma membrane GLUT4 and GLUT1 levels in GLUT4myc- and GLUT1myc-overexpressing cells, respectively. RA did not cause a significant increase in plasma membrane GLUT4myc levels (117 ± 2.70% of control, *p* > 0.05) (Figure 8A). On the other hand, maximal insulin (10^−7^ M, 20 min) and metformin (1 mM for 2 h) treatment stimulated GLUT4myc translocation (207 ± 7.86% of control and 175 ± 12.47% of control, respectively, both *p* < 0.001) (Figure 8A). RE did not have any effect on GLUT4myc translocation (106 ± 3.20% of control, *p* > 0.05). Similarly, RA and RE did not cause a significant increase in plasma membrane GLUT1myc levels (116 ± 2.51% of control and 113 ± 5.52% of control, respectively, both *p* > 0.05) (Figure 8B). Insulin (10^−7^ M, 20 min) and metformin (1 mM, 2 h) significantly increased plasma membrane GLUT1 levels (139 ± 1.44% of control and 135 ± 2.14% of control, respectively, both *p* < 0.001) (Figure 8B). It should be noted that RA increased glucose uptake in GLUT4myc (178 ± 8.62% of control, *p* < 0.01) and GLUT1myc (198 ± 3.84% of control, *p* < 0.001) overexpressing cells (Figure 8C,D).

## 3. Discussion

We previously found that treatment of L6 muscle cells with 5 μg/mL of rosemary extract increased glucose uptake to a level comparable to maximum stimulation observed with insulin and metformin [27], and we hypothesized that specific polyphenols present in the RE may contribute to the increase in glucose uptake. We measured the direct effect of carnosic acid on muscle cells and found a significant increase in glucose uptake and activation of AMPK [31]. In the current study, we continued our investigation and focused on the rosemary polyphenol rosmarinic acid (RA).

To this end, we conducted high-performance liquid chromatography (HPLC) to measure the levels of RA present in our RE, and it was found to be 13.39%. This concentration of RA was comparable to previous concentrations reported to be found in RE [42]. Based on the values obtained from the HPLC and the molecular weight of RA (MW: 360.32 g/mol), we calculated that in media containing 5 μg/mL of our rosemary extract, the corresponding concentration of RA is 2 μM. We wished to examine if this micromolar concentration of RA affects muscle glucose uptake. This study is the first to demonstrate that RA concentration as low as 2 μM stimulated glucose uptake in L6 muscle cells to significant levels. Importantly, a stimulation comparable to maximal insulin and metformin was observed at 5 μM RA, indicating a potential for RA to be used similar to metformin as a pharmacological intervention in the treatment of insulin resistance.

RA did not affect the maximal (100 nM) but significantly enhanced the submaximal (3 nM) insulin response. These data suggest that when the insulin response is reduced (is at submaximal levels), as in the case of insulin resistance, treatment with RA has the potential to restore glucose uptake and therefore may be beneficial. Future studies should examine the effects of RA on insulin-resistant muscle cells.

In the present study, it was found that the effect of RA on glucose uptake is PI3K-independent, since wortmannin, an irreversible inhibitor of PI3K, did not affect the RA-stimulated glucose uptake. Wortmannin completely blocked the insulin-stimulated glucose uptake, and therefore, we are confident that wortmannin effectively blocked PI3K activation in our studies. Additionally, Akt phosphorylation and phosphorylation of its downstream target, p70 S6K, was not affected by RA, while a robust activation was seen with insulin treatment. These data clearly indicate activation of the PI3K–Akt cascade by insulin without any effect on this cascade by RA.

Importantly, treatment with RA significantly increased AMPK phosphorylation at its Thr 172 residue, which correlates highly with kinase activity and is used as a marker of its activation [8,9,14,32]. Moreover, treatment with RA significantly increased the phosphorylation of ACC, a downstream effector of AMPK, used as an index of AMPK activity in numerous studies [8,9,14,32]. Compound C, an ATP-competitive inhibitor of AMPK, significantly decreased the RA-mediated glucose uptake, indicating AMPK involvement in the action of RA. Compound C abolished the RA-stimulated phosphorylation of AMPK and ACC, indicating effective inhibition of AMPK in our study. 

Ca^2+^/calmodulin-dependent protein kinase kinase (CaMKK), an upstream regulator of AMPK, phosphorylates Thr172 and increases AMPK activity [43,9,14]. CaMKK may be mediating the increase of muscle AMPK activity by a variety of stimuli including contraction [44] and oxytocin [45]. We hypothesized that CaMKK may be involved in RA-stimulated glucose uptake, and to address our hypothesis we used STO-609, an established inhibitor of CaMKK [33]. Our study showed that inhibition of CaMKK by STO-609 did not affect the RA-stimulated glucose uptake or the RA-induced AMPK phosphorylation, indicating that this kinase is not involved in the mechanism of action of RA. According to Hawley et al., there is a possibility that STO-609 may directly inhibit AMPK activity without affecting the phosphorylation of AMPK [46]. Therefore, we examined phosphorylation of ACC, the downstream target of AMPK, established as a proxy of AMPK activity. STO-609 did not have any effect on the RA-induced phosphorylation of ACC, further indicating that CaMKK is not involved in the RA-stimulated glucose uptake.

It is important to note that the RA-stimulated glucose uptake, although significantly reduced by compound C, was not completely abolished. This partial inhibition of RA-stimulated glucose uptake under conditions of AMPK inhibition suggests that signaling molecules other than AMPK may be involved in the action of RA. Different studies indicate that conventional/novel PKCs may be involved in the increase of muscle glucose uptake by different stimuli [34,47,48]. A significant part of metformin-stimulated muscle glucose uptake was found to be independent of AMPK and dependent on novel /conventional PKCs [34]. We therefore hypothesized that PKCs may be involved in the RA-stimulated glucose uptake, and to address our hypothesis, we used bisindolylmaleimide I (BMD), a selective inhibitor of conventional/novel PKCs widely used in other studies [37,49], and found that the RA-stimulated glucose uptake was not affected by BMD. We further performed western blot experiments examining phosphorylation/activation of PKCs. We used an antibody that recognizes endogenous levels of conventional (α, β_I_, β_II_) and novel (δ, ε, η and θ) PKCs phosphorylated at the carboxy-terminal residue homologous to Ser 660 of PKCβ_II_ (P-PKC). It should be noted that PKC phosphorylation at this specific site recognized by the antibody used correlates with increased PKC activity [41]. We are confident that PKCs are expressed and can be activated in L6 myotubes, as we found a robust phosphorylation/activation of PKC with 12-*O*-tetradecanoylphorbol-13-acetate (TPA) treatment. Importantly, the TPA-induced PKC phosphorylation/activation was completely abolished by BMD, indicating that in our studies, BMD was an effective inhibitor of PKCs. Taken together, our data indicate that the RA-stimulated glucose uptake is independent of conventional and novel PKCs. It is possible that atypical PKCs may be involved in the RA-induced glucose uptake and this could be further examined in the future.

L6 muscle cells express GLUT1, GLUT3 and GLUT4 glucose transporters [50]. We measured plasma membrane GLUT1 and GLUT4 levels in L6 cells overexpressing GLUT1 or GLUT4, respectively. Our data showed that there was no significant increase in plasma membrane levels of GLUT1 or GLUT4 by RA, although there was a significant increase in glucose uptake. RE treatment also did not significantly increase plasma membrane levels of GLUT1 or GLUT4, in agreement with our previous studies [27,31]. On the contrary, both insulin and metformin increased GLUT1 and GLUT4 plasma membrane levels in agreement with previous studies [27,31,51]. It is possible that the increase in glucose uptake by RA in the present study may be due to either GLUT3 translocation (since GLUT3 is also expressed in L6 cells [50]) or increased intrinsic activity of the plasma membrane-localized GLUTs. A recent study has shown that acute (30 min) treatment with triiodothyronine (T3) increased glucose uptake in GLUT4myc-overexpressing L6 muscle cells without increasing GLUT4 translocation to the cell membrane [52], suggesting that T3 enhanced the activity of the GLUTs present on the cell membrane. The effect of RA is similar to the effect observed with RE [27] and CA [31] treatment in our previous studies, and similar to the effect of T3 [52], suggesting that these stimuli/treatments may increase the activity of plasma membrane-localized GLUTs. 

A limited number of studies performed in cardiomyocytes [53,54] and C_2_C_12_ muscle cells [55] indicate that RA reduces cellular reactive oxygen species (ROS) levels and has antioxidant properties. Antioxidants such as astaxanthin, α-tocopherol and α-lipoic acid [56] have been shown to increase glucose uptake in L6 muscle cells, and therefore, it is possible that the increase in glucose uptake seen by RA in our study is due to its antioxidant properties. In a recent study, treatment of L6 muscle cells with 20 μM RA prevented the palmitate-induced decline of the mitochondria biogenesis markers PGC-1a, SIRT-1 and TFAM, an effect that was similar to metformin treatment [57]. In addition, RA prevented the palmitate-induced IRS-1 (ser307) phosphorylation and the decline in plasma membrane GLUT4 levels [57].

A limited number of studies have also examined the antidiabetic effects of RA in vivo. Administration of RA (120–200 mg/kg, 7 days) dose-dependently ameliorated hyperglycemia and insulin resistance and prevented reduction in GLUT4 expression in streptozotocin (STZ)-induced diabetic rats [26]. Furthermore, RA (100 mg/kg bw/day) increased AMPK and modulated the expression of mitochondrial biogenesis markers SIRT-1, PGC-1a and TFAM in high-fat-diet STZ-diabetic rats in addition to restoring GLUT4 levels to the plasma membrane [57].

It is important to note that for the first time, the tolerability and safety of RA was recently assessed in humans [58]. Healthy volunteers were given *Melissa officinalis* (lemon balm) extract containing 500 mg of RA. The total serum concentration of RA reached maximum (162.20 nM) in 1 h after the administration in fasting state [58], clearly indicating that RA is readily absorbed. More importantly, administration of the extract containing RA had no effect on the blood, kidney or liver function parameters, and no side-effects were reported [58]. Although this study indicates RA is well-tolerated and safe in humans [58], more studies are required to examine safety and potential toxicity of chronic RA administration.

## 4. Conclusions

In conclusion, the present study shows a direct effect of rosmarinic acid (RA) to significantly increase glucose uptake in L6 muscle cells to levels comparable to insulin and metformin. The glucose uptake was partially but significantly inhibited in the presence of compound C, an inhibitor of AMPK, but was not affected by wortmannin, an inhibitor of PI3K, indicating a mechanism that is partially dependent on AMPK and independent of PI3K. Overall, more studies should be performed to investigate further the potential of rosmarinic acid to be used to prevent and/or manage insulin resistance and T2DM.

## 5. Materials and Methods

### 5.1. Materials

Fetal bovine serum (FBS), rosmarinic acid, dimethyl sulfoxide (DMSO), *o*-phenylenediamine dihydrochloride (OPD), cytochalasin B and STO-609 inhibitor were purchased from Sigma Life Sciences (St. Louis, MO, USA). Materials for cell culture were purchased from GIBCO Life Technologies (Burlington, ON, Canada). Several antibodies were used in our study, including phospho- and total-AMPK (CAT 2531 and 2532, respectively, New England BioLabs (NEB) (Missisauga, ON, Canada), rabbit, 1:1000 dilution), Akt (CAT 9271 and 9272, New England BioLabs (NEB) (Missisauga, ON), rabbit, 1:1000 dilution), ACC (CAT 3661 and 3676, New England BioLabs (NEB) (Missisauga, ON), rabbit, 1:1000 dilution), p70 S6K (CAT 9205 and 2708, New England BioLabs (NEB) (Missisauga, ON), rabbit, 1:1000 dilution), phospho-PKC (pan) (βII Ser660) (CAT 9371, New England BioLabs (NEB) (Missisauga, ON), rabbit, 1:1000 dilution), 12-*O*-tetradecanoylphorbol-13-acetate (TPA) (CAT 4174, New England BioLabs (NEB) (Missisauga, ON)) and HRP-conjugated anti-rabbit antibodies (CAT 7074, New England BioLabs (NEB) (Missisauga, ON), 1:2000 dilution). In addition, anti-c-myc antibodies (CAT 3956, Sigma Life Sciences, 1:500 dilution) and peroxidase-conjugated goat anti-rabbit IgG were purchased from Sigma Life Sciences and Jackson ImmunoResearch Labs (West Grove, PA, USA (CAT: 111-035-144, 1:1000 dilution), respectively. Insulin (Humulin R) was from Eli Lilly (Indianapolis, IN, USA). Compound C, wortmannin and bisindolylmaleimide I were purchased from Calbiochem (Gibbstown, NJ, USA). Luminol Enhancer reagents, polyvinylidene difluoride (PVDF) membrane, and reagents for electrophoresis and Bradford protein assay were purchased from BioRad (Hercules, CA, USA). [^3^H]-2-deoxy-d-glucose was purchased from PerkinElmer (Boston, MA, USA).

### 5.2. Measurement of Rosmarinic Acid Levels in Rosemary Extract

The preparation of RE was conducted as previously described. As a standard we used RE and RA which were dissolved at a concentration of 2 mg/mL in sterile DMSO. 2.5 μL of previously prepared aliquots were injected into a reversed-phase Agilent 1100 series high-performance liquid chromatography (HPLC) instrument which was equipped with an autosampler. Quantification and separation were conducted at 25 °C via mobile phase consisting of solvent A and solvent B. Solvent A consisted of 0.1% formic acid and solvent B consisted of acetonitrile. Over 30 min, a linear gradient of 95% solvent A and 5% solvent B was attained. The detection was set at 254 nm and the flow rate at 0.4 mL/min. Identification of RA was achieved by comparison of the actual retention time to those of authentic reference standard of RA. Furthermore, quantification of RA was done by calculating the peak areas after completion of HPLC separation. Mean total content was expressed in % (g/100 g dry weight extract).

### 5.3. Cell Culture, Treatment and Glucose Uptake Assay

L6 rat muscle (parental, overexpressing GLUT4myc and GLUT1myc) cells were grown in α-MEM media containing 2% *v*/*v* fetal bovine serum (FBS) until fully differentiated. Myotube stage was reached at approximately 6 to 7 days after seeding. Prior to the experiments, the cells were serum-deprived for 3 h followed by treatment as described in the figures. After treatment, the cells were washed with HEPES-buffered saline (HBS) solution and exposed to 10 µM [^3^H]-2-deoxy-d-glucose in HBS for 10 min to measure glucose uptake. Exposure to 10 µM cytochalasin B was used to determine the non-specific glucose uptake. At the end of the 10 min uptake time, the cells were rinsed with 0.9% NaCl solution followed by 0.05 M NaOH lysis buffer. Liquid scintillation counter (PerkinElmer) was used to measure radioactivity. Bio-Rad protein assay was used to quantify protein levels.

### 5.4. Immunoblotting

After treatment, whole lysates were obtained by washing the cells with ice-cold HBS solution and lysing with ice-cold lysis buffer. Lysates were kept at −20 °C. Protein samples (15 µg) were separated using sodium dodecyl sulfate polyacrylamide gel electrophoresis (SDS-PAGE) and transferred to PVDF membrane followed by exposure to blocking buffer containing 5% *w*/*v* dry milk powder in Tris-buffered saline and overnight incubation with the primary antibody at 4 °C followed by exposure to HRP-conjugated anti-rabbit antibody for 1 h and Luminol Enhancer reagents (BioRad). The corresponding bands were visualized using FluroChem software (ThermoFisher, Waltham, MA, USA).

### 5.5. GLUT4myc and GLUT1myc Translocation Assay

After treatment, fully differentiated GLUT4myc- or GLUT1myc-overexpressing myotubes were fixed using 3% paraformaldehyde for 10 min at 4 °C and additional 20 min at room temperature followed by exposure to 1% glycine in PBS, and blocked with 10% goat serum in PBS for 15 min at 4 °C. The cells were then incubated with an anti-myc antibody for 60 min at 4 °C followed by exposure to peroxidase-conjugated goat anti-rabbit IgG at room temperature for 30 min. The cells were rinsed and incubated with *o*-phenylenediamine dihydrochloride (OPD) reagent for 30 min at room temperature and the reaction was stopped by adding 3 M HCl. The absorbance of the supernatant was measured at 492 nM.

### 5.6. Statistical Analysis

GraphPad Prism v7.0 (GraphPad Software, Inc. La Jolla, CA, USA) was used to calculate the significance of the differences between groups using ANOVA followed by Tukey’s post-hoc analysis. Statistical significance was assumed at *p* < 0.05.
